# Cost-effectiveness of analysis serplulimab plus chemotherapy as first-line therapy for PD-L1-positive advanced esophageal squamous cell carcinoma

**DOI:** 10.3389/fonc.2023.1216960

**Published:** 2023-10-31

**Authors:** Hanrui Zheng, Jiafeng Li, Feng Wen, Na Su

**Affiliations:** ^1^ Department of Pharmacy, West China Hospital, Sichuan University, Chengdu, China; ^2^ West China School of Pharmacy, Sichuan University, Chengdu, China; ^3^ Mental Health Center, West China Hospital, Sichuan University, Chengdu, China; ^4^ Department of Medical Oncology, Cancer Center, West China Hospital, Sichuan University, Chengdu, China; ^5^ West China Biomedical Big Data Center, Sichuan University, Chengdu, China; ^6^ Med-X Center for Informatics, Sichuan University, Chengdu, China

**Keywords:** cost-effectiveness, serplulimab, chemotherapy, esophageal squamous cell carcinoma, programmed cell death protein 1

## Abstract

**Objective:**

Our study aimed to evaluate the cost-effectiveness of the addition of serplulimab to chemotherapy (cisplatin and fluorouracil) for programmed death-ligand 1 (PD-L1) positive advanced esophageal squamous cell carcinoma (ESCC) as the first-line treatment in China.

**Methods:**

A three-state Markov model was established to assess the incremental cost-effectiveness ratio (ICER) for serplulimab plus chemotherapy versus chemotherapy alone. Survival data were extrapolated from the ASTRUM-007 trial, cost data were derived from local sources, and utilities were derived from published literature. Health outcomes were measured as quality-adjusted life-years (QALYs). Sensitivity and probability sensitivity analyses were used to investigate the robustness of the model.

**Results:**

In the base-case analysis, compared with chemotherapy alone, serplulimab gained an additional 0.16 QALYs with an incremental cost of $29,547.88, leading to an ICER of $184,674.25/QALY. Additionally, the subgroup analyses presented that the ICERs of serplulimab plus chemotherapy were $157,892.50/QALY and $127,996.45/QALY in advanced ESCC patients with 1≤ CPS< 10 and CPS≥ 10, respectively. These ICERs significantly exceeded the Chinese willingness-to-pay (WTP) threshold. The deterministic sensitivity analysis illustrated that the cost of progression-free survival in serplulimab plus chemotherapy group was the parameter with the strongest influence on the ICERs.

**Conclusion:**

In the Chinese health care system, with 3 times China’s per capita gross domestic product as the WTP threshold, compared with chemotherapy alone, serplulimab combined chemotherapy is not economical for PD-L1-positive advanced ESCC in the first-line setting.

## Introduction

Esophageal cancer is the seventh most frequent cancer globally, with more than 604,000 new cases identified each year (1). Additionally, esophageal cancer is the sixth greatest cause of cancer-related death, accounting for approximately 500,000 cancer-related deaths each year. Meanwhile, the overall 5-year survival rate for esophageal cancer patients is less than 20% (1). The most common histologic subtype of esophageal cancer in China is esophageal squamous cell carcinoma (ESCC) (2). Because early ESCC does not have specific clinical symptoms, most patients who do not receive an early diagnosis are often diagnosed at an advanced stage, resulting in a poor quality of life and a poor prognosis (3).

Chemotherapy is the first-line treatment for advanced or metastatic ESCC, and the most commonly used chemotherapy drugs for esophageal cancer include cisplatin, 5-fluorouracil (5-FU), and doxorubicin (4). Second-line chemotherapy is usually performed using a single drug, such as docetaxel or paclitaxel (5). However, the significant toxicity of chemotherapy, including nephrotoxicity, neurotoxicity, ototoxicity, and bone marrow suppression, as well as drug resistance, has a negative impact on its clinical use (6).

Immune checkpoint inhibitors have been suggested as a first-line treatment option for advanced ESCC patients in recent years. Pembrolizumab or nivolumab, in combination with chemotherapy, produced encouraging results in the treatment of advanced ESCC cancer (7, 8). Subsequently, China’s domestic immunotherapy drugs, including sintilimab, camrelizumab, toripalimab and tislelizumab, have also achieved promising results in advanced ESCC (9–12). There is no doubt that these novel drugs offer more treatment options for advanced ESCC patients.

Recently, a randomized phase III trial (ASTRUM-007) in China investigated chemotherapy with the programmed cell death protein-1 (PD-1) inhibitor serplulimab for PD-L1-positive advanced ESCC patients. The combination of serplulimab plus chemotherapy every two weeks improved progression-free survival (PFS) and overall survival (OS) with acceptable toxicity (median PFS, 5.8 vs. 5.3 months; hazard ratio (HR) = 0.60; p< 0.0001; median OS, 15.3 vs.11.8 months; HR = 0.68; p = 0.0020) (13). For advanced ESCC patients with PD-L1 combination positive score (CPS) ≥10, the benefit was even greater in the serplulimab plus chemotherapy group compared with chemotherapy alone group (median PFS, 7.1 vs. 5.3 months; HR = 0.60; p< 0.0001; median OS, 18.6 vs.13.9 months; HR = 0.59; p = 0.0082).

Despite its efficacy, serplulimab is much more expensive than the existing standard chemotherapy, which might impose a considerable financial strain on the national healthcare system. This is especially important because serplulimab is scheduled to be licenced by China’s National Medical Products Administration (NMPA). To further understand the relative costs of the two therapies on health outcomes, an economic comparison of serplulimab versus conventional chemotherapy as first-line therapy for advanced ESCC is necessary. The goal of our study is to compare the cost-effectiveness of serplulimab plus chemotherapy with chemotherapy alone for untreated, PD-L1-positive advanced ESCC patients in the Chinese healthcare system.

## Methods

### Patients and interventions

The target population and treatment strategies are based on the ASTRUM-007 (NCT03958890) clinical study, which is a randomized, double-blind, multicentre, phase III clinical study (13). Previously untreated locally advanced or metastatic ESCC patients with PD-L1 CPS≥ 1 were randomly assigned (2:1) to receive treatment with serplulimab plus chemotherapy or chemotherapy alone. This clinical trial was conducted in China and included a total of 976 advanced ESCC patients. Serplulimab (3 mg/kg D1) was given in conjunction with cisplatin (50 mg/m^2^ D1) and a continuous infusion of 5-FU (1,200 mg/m^2^, D1-2) every two weeks. Only cisplatin and 5-FU were used in the chemotherapy arm. The serplulimab plus chemotherapy group had a median follow-up of 14.9 months, and the chemotherapy group 15.0 months.

### Model structure

**Figure 1:**
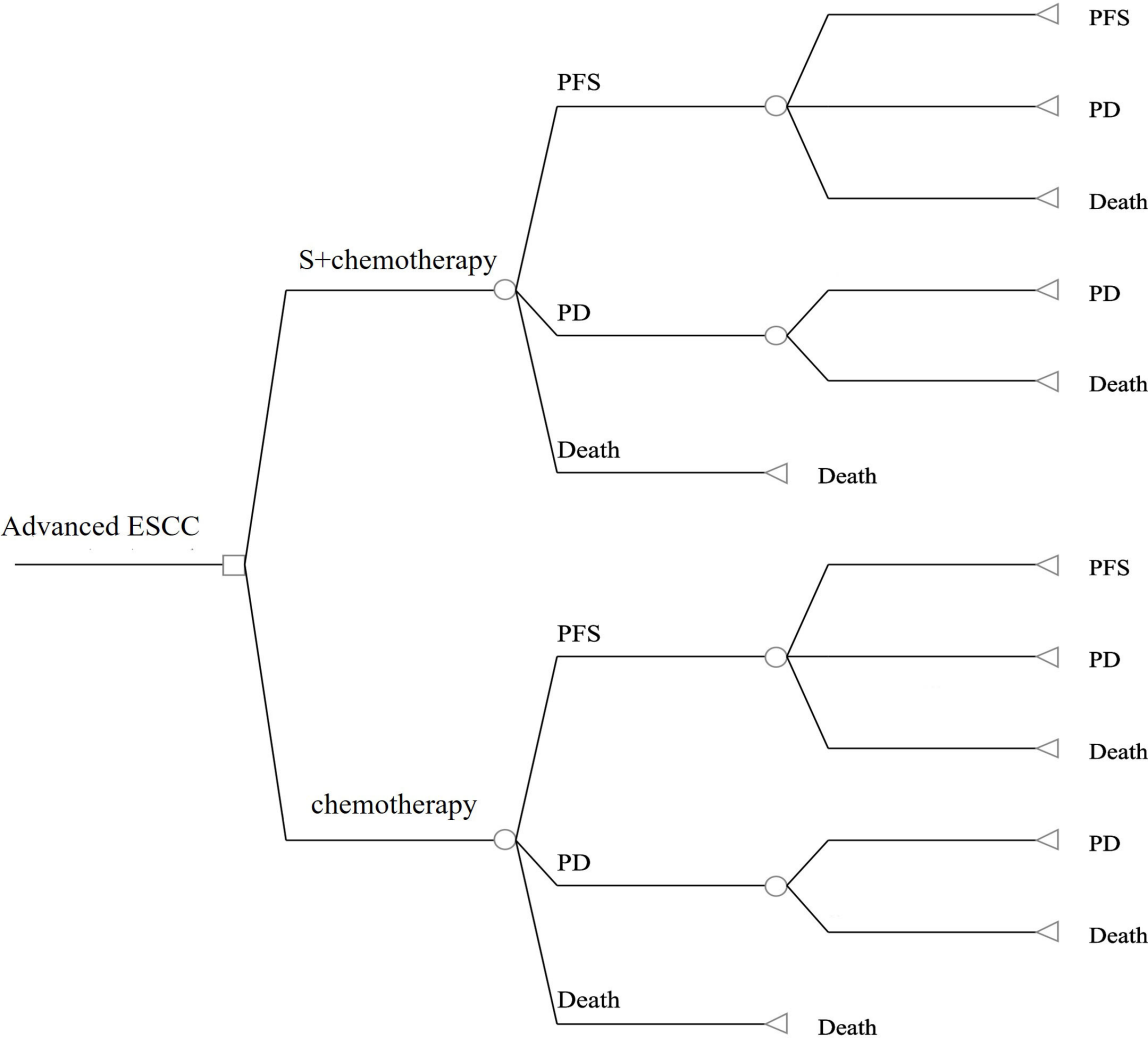
The structure of our model in the first-line setting for advanced esophageal squamous cell carcinoma. S, serplulimab; ESCC, esophageal squamous cell carcinoma;PFS, progression-free survival; PD, progressive disease.

**Figure 2:**
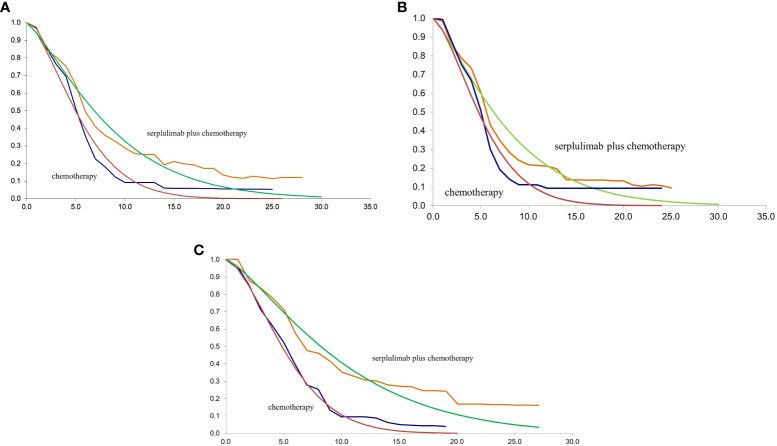
**(A)** The fitting of PFS in Weibull curves for patients with PD-L1 CPS ≥ 1; **(B)** The fitting of PFS in Weibull curves for patients with PD-L1 1≤CPS<10; **(C)** The fitting of PFS in Weibull curves for patients with PD-L1 CPS ≥ 10; CPS, combined positive score; PFS, progression-free survival.

**Figure 3:**
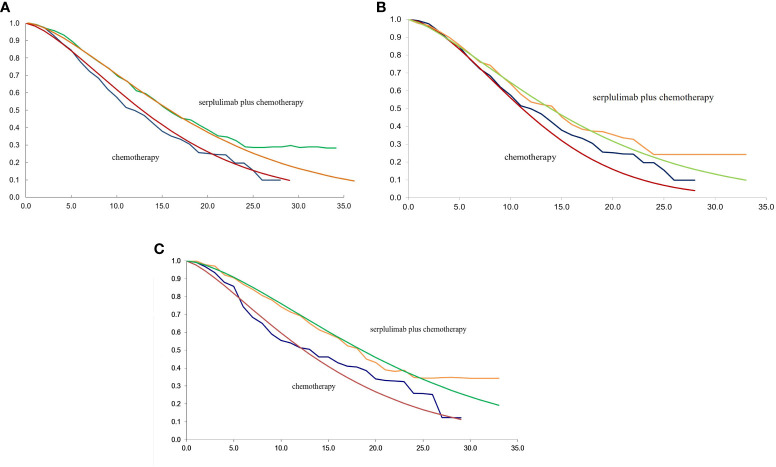
**(A)** The fitting of OS in Weibull curves for patients with PD-L1 CPS ≥ 1; **(B)** The fitting of OS in Weibull curves for patients with PD-L1≤CPS<10; **(C)** The fitting of OS in Weibull curves for patients with PD-L1 CPS ≥ 10; CPS, combined positive score; OS, overall survival.


The TreeAge Pro 2020 was used to create the Markov model in this study (Williamstown, MA). According to the development of advanced ESCC, three mutually exclusive health states, including PFS, progressive disease (PD), and death were defined in this model, as shown in **Figure 1**. We assumed that at the start of the simulation, all patients were in the PFS state and that transitions between states could occur based on the transition probabilities. In other words, patients in the PFS state could either remain in the PFS state or transition to the PD or death stage. Patients in the state of PD could either stay in PD state or transition to death. The GetData Graphical Digitizer was used to obtain Kaplan-Meier survival results for PFS and OS from the ASTRUM-007 study (version 2.26). We reconstructed individual patient data following the method of Hoyle et al. (14). Using the R statistical program, the Weibull distribution was fitted for the PFS and OS curves (version 4.0.5) (**Figures 2**, **3**). The shape parameter (γ) and scale parameter (λ) of Weibull distribution were used to calculate the transfer probabilities. Transition probabilities (P) in our model were obtained from the following formula:

$$\text{P}({\text{t}}_{\text{u}})=1-\exp\{\text{λ}{\left(\text{t}-\text{u}\right)}^{\text{γ}}-{\text{λt}}^{\text{γ}}\}\;(\text{λ}>0,\text{γ}>0)$$

where u is the Markov cycle and t_u_ represents the arrival at state t after u Markov cycles (15). The Markov cycle length was 1 month, and the time horizon in the model was set to 10 years. The parameters used in the model are illustrated in **Table 1**. The outcomes of our model were quality-adjusted life-years (QALYs). The willingness-to-pay (WTP) was established at $37,663.26/QALY based on a recommendation of three times the gross domestic product (GDP) of China (16). The incremental cost-effectiveness ratio (ICER) and the incremental monetary benefit (INMB) were calculated based on the following formulas:

**Table 1:** Major parameters in the Markov model.

Parameter	Serplulimab+ chemotherapy	Chemotherapy	Distribution
Costs per cycle ($)			
Serplulimab	3,415.47	NA	Gamma
Chemotherapy	705.15	578.75	Gamma
Antiemetic drugs	200.16	164.28	Gamma
Tests	384.63	330.37	Gamma
AEs	50.13	40.86	Gamma
Hospitalization	40.34	33.11	Gamma
Subsequent therapy cost	430.95	869.24	Gamma
Utilities			
PFS	0.68	0.68	Beta
PD	0.42	0.42	Beta
Survival data			
Patients with CPS ≥1			
Weibull model in PFS	λ=0.0594	λ=0.0595	Fixed
	γ=1.275	γ=1.532	Fixed
Weibull model in OS	λ=0.0119	λ=0.0158	Fixed
	γ=1.478	γ=1.482	Fixed
Patients with 1≤CPS<10			
Weibull model in PFS	λ=0.0678	λ=0.0655	Fixed
	γ=1.258	γ=1.513	Fixed
Weibull model in OS	λ=0.0176	λ=0.0125	Fixed
	γ=1.396	γ=1.667	Fixed
Patients with CPS ≥10			
Weibull model in PFS	λ=0.0456	λ=0.0228	Fixed
	γ=1.294	γ=1.354	Fixed
Weibull model in OS	λ=0.0086	λ=0.0536	Fixed
	γ=1.504	γ=1.619	Fixed

PFS, progression-free survival; PD, progressive disease; AEs, adverse events; CPS, combined positive score; NA, not applicable.


ICER=ΔCost/ΔEffectiveness

INMB=ΔEffectiveness×WTP−ΔCost

$$\begin{array}{c} \Delta \text{Effectiveness}=\text{Effectiveness of serplulimab plus chemotherapy}\\ -\text{Effectiveness of chemotherapy} \end{array}$$

ΔCost=Costs of serplulimab plus chemotherapy−Costs of chemotherapy

When the ICER is less than the threshold, the additional cost is deemed acceptable; when the ICER exceeds the threshold, the increased cost is deemed unjustifiable. When the INMB > 0, the intervention is considered to be a cost-effectiveness option.

### Cost and utility estimates

Only direct medical expenditures were considered, including medicine costs (serplulimab, chemotherapy drugs, antiemetics, and so on), blood and biochemical test costs, imaging examination costs, treatment-related adverse event (AE) costs, and subsequent treatment costs. The chemotherapy dosages were determined using the standard body surface area and standard weight (1.72 m^2^, 65 kg) (17). Prices were derived from the West China Hospital since drug prices are approximately the same in most Chinese medical centres. All costs were converted into US dollars at the current conversion rate of $1 = ¥ 6.77. For second-line treatment, the ASTRUM-007 trial reported that 139 patients in the serplulimab plus chemotherapy group and 95 in the placebo plus chemotherapy group received subsequent anticancer therapy. Additionally, 64 patients in the serplulimab plus chemotherapy group and 61 patients in the placebo plus chemotherapy group received immunotherapy. For other patients, we hypothesized that the following treatment was chemotherapy. Base on the Chinese Society of Clinical Oncology (CSCO) guidelines, irinotecan and docetaxel were used for them. Utility is a measure of patient quality of life that ranges from 0 to 1. The values for health utility were derived from recently published literature. The utility value for the PFS, PD, and death states was 0.68, 0.42, and 0, respectively (18). Both the costs and utilities were discounted at a rate of 5% per year.

### Subgroup analysis

In the subgroup analysis, we also analyzed the cost-effectiveness of serplulimab plus chemotherapy for advanced ESCC patients with PD-L1 1 ≤CPS<10 and PD-L1 CPS ≥10. The clinical information of patients with PD-L1 1 ≤CPS<10 and PD-L1 CPS ≥10 was derived from the Kaplan–Meier survival curves reported in the ASTRUM-007 trial.

### Sensitivity analysis

The stability of the model was examined using sensitivity analyses. A one-way probabilistic sensitivity analysis was conducted to assess the influence of model uncertainty on the cost-effectiveness of treatment strategies. Parameters in the model were changed within ±20% of the baseline value to examine their impact on the ICERs and INMB. A tornado diagram was used to show the results of the one-way sensitivity analysis. We employed a probabilistic sensitivity analysis to simultaneously investigate the uncertainty of all model parameters through 1000 iterations of a Monte Carlo simulation.

## Results

### Base case results

**Table 2** presents the results of basic analysis of the two treatment regimens. Serplulimab produced an incremental 0.16 QALYs, with an incremental cost of $ 29,547.88, which led to an ICER of $184,674.25/QALY, significantly exceeding the Chinese WTP threshold ($37,663.26/QALY). The INMB was -$29,547.88 at a WTP threshold of $37,663.26/QALY. The subgroup demonstrated that the combined serplulimab and chemotherapy group provided a gain of 0.18 QALYs at additional cost of $28,420.65 compared to the chemotherapy alone group for advanced ESCC patients with PD-L1 expression level of 1 ≤CPS<10. The combined serplulimab and chemotherapy group provided a gain of 0.29 QALYs with incremental costs of $37,118.97 compared to the chemotherapy alone group for advanced ESCC patients with PD-L1 expression level of CPS≥10. The ICER was $157,892.50 and $127,996.45 per QALY in patients with PD-L1 1≤CPS<10 and CPS≥ 10, respectively. These ICERs also exceeded the WTP threshold. Therefore, the addition of serplulimab to chemotherapy is not an economical choice for advanced ESCC patients with PD-L1-positive, PD-L1 1≤ CPS< 10 and CPS ≥ 10 in China.

**Table 2:** Results of the cost-effectiveness analysis.

Result	Serplulimab+chemotherapy	Chemotherapy
Patients with CPS ≥1		
Cost ($)	43,427.43	13,879.55
Incremental costs ($)	29,547.88	NA
Effectiveness (QALYs)	0.78	0.62
Incremental effectiveness (QALYs)	0.16	NA
ICER ($/QALY)	184,674.25	NA
INMB ($)	-29,547.88	NA
Patients with 1≤CPS<10		
Cost ($)	40,318.56	11,897.91
Incremental costs ($)	28,420.65	NA
Effectiveness (QALYs)	0.72	0.54
Incremental effectiveness (QALYs)	0.18	NA
ICER ($/QALY)	157,892.50	NA
INMB ($)	-28,420.65	NA
Patients with CPS ≥10		
Cost ($)	51,021.86	13,902.89
Incremental costs ($)	37,118.97	NA
Effectiveness (QALYs)	0.91	0.62
Incremental effectiveness (QALYs)	0.29	NA
ICER ($/QALY)	127,996.45	NA
INMB ($)	-26,196.62	NA

CPS, combined positive score; QALY, quality-adjusted life year; ICER, Incremental cost-effectiveness ratio; INMB, incremental net monetary benefit; NA, not applicable.

### Sensitivity analyses

#### One-way sensitivity analysis

**Figure 4:**
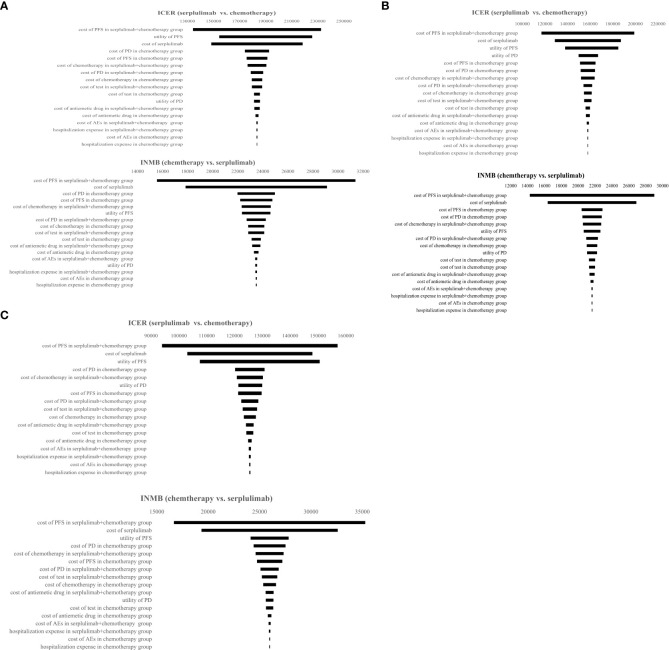
Tornado diagram representing results of one-way sensitivity analysis. It presents influence of important variables on the ICERs and INMB. **(A)** Patients with PD-L1 CPS ≥ 1; **(B)** Patients with PD-L1≤CPS<10; **(C)** Patients with PD-L1 CPS ≥ 10. PFS, progression-free survival, PD, progressive disease; AEs, adverse events; ICER, incremental cost-effectiveness ratio; INMB, incremental net monetary benefit.

The results of the one-way sensitivity analysis are presented in a tornado diagram (**Figure 4**). When the model parameters were changed within a certain range, the cost of PFS in serplulimab and chemotherapy had the greatest impact on the model results regardless of PD-L1 expression level. The utility of PFS and the price of serplulimab also had a considerable impact on ICERs and INMB. In addition, hospitalization expenses and AEs management costs had a weak influence on the results.

#### Probabilistic sensitivity analysis

**Figure 5:**
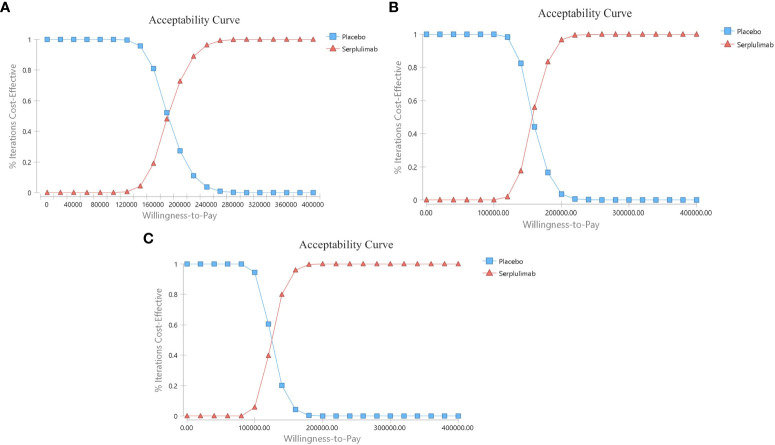
The cost-effectiveness acceptability curves. **(A)** Patients with PD-L1 CPS ≥ 1; **(B)** Patients with PD-L1≤CPS<10; **(C)** Patients with PD-L1 CPS ≥ 10.

The probabilistic sensitivity analysis results are shown in **Figure 5**. When the WTP threshold was 3 times the GDP per capita in China, the cost-effectiveness acceptability curve showed a 0% probability of serplulimab combined with chemotherapy compared to chemotherapy alone is a cost-effective strategy in the overall population and in subgroups. The acceptable proportion of the serplulimab combined with chemotherapy group was increased as the WTP threshold increased.

## Discussion

According to the Global Burden of Disease (GBD) visualization database, the number of patients diagnosed with esophageal cancer in China grew from 254,169 in 1990 to 498,410 in 2019, accounting for half of all cases worldwide (19). The tremendous rise in morbidity and death has placed significant social and economic strain on the Chinese healthcare system (20). According to certain estimations, the total annual direct medical spending for esophageal cancer in China will rise by 128.7% between 2013 and 2030 (from 33.4 billion US dollars to 76.4 billion US dollars) (21). Therefore, it is urgent to conduct economic evaluations of drugs for esophageal cancer to optimize the allocation of medical resources.

Immunotherapy has transformed the treatment of advanced ESCC, and researchers have emphasized the benefits of PD-1/PD-L1 inhibitors. Serplulimab combined with chemotherapy significantly improved PFS and OS in PD-L1-positive advanced ESCC patients, especially those with PD-L1 CPS ≥10. However, the high cost of cancer medications may limit their broad usage. Given the economic burden of advanced ESCC, determining the cost-effectiveness of innovative therapies is a prerequisite to providing patients with treatment alternatives.

This was the first cost-effectiveness analysis of serplulimab in the first-line treatment for PD-L1-positive advanced ESCC patients in China using a Markov model. Our base case results showed that the ICER of serplulimab combined with chemotherapy was $18,4674.25/QALY, which was higher than the WTP threshold. Serplulimab combined with chemotherapy was a favorable option compared to chemotherapy with a WTP threshold of $200,000/QALY, and there was no such advantage at a WTP threshold of $100,000/QALY. Therefore, serplulimab combined with chemotherapy may not be a cost-effective option in China PD-L1-positive advanced ESCC patients.

Several studies have analyzed the cost-effectiveness of PD-1 inhibitors in advanced esophageal cancer. Nivolumab plus ipilimumab or chemotherapy is not cost-effective as a first-line treatment for advanced esophageal cancer in China (22). From an economic perspective in China, the addition of pembrolizumab to chemotherapy for esophageal cancer is not economical, regardless of PD-L1 expression (23). However, a domestic monoclonal antibody, sintilimab, in combination with chemotherapy has been suggested to be a cost-effective option for Chinese advanced ESCC patients, with two other studies reporting similar results (24, 25). The main reason is that the price of these drugs has a great impact on their ICERs. Sintilimab was added to the National Reimbursement List of Drug Negotiation (NRDL) in 2019, thus enhancing its affordability. The price of sintilimab has been reduced by approximately 60% through negotiation.

As one of the countries with the least affordable anticancer prices worldwide, China has made vital efforts to enhance accessibility. One such commendable initiative is the implementation of the National Reimbursement Drug List (NRDL) policy. The prices of anticancer drugs, especially innovation drugs, was decreased by government reimbursement negotiation, thereby alleviating the national financial burden (26). The number of anticancer drugs included in the NRDL has been steadily increasing, resulting in improved patient access over the past four years. It is anticipated that this reform will persist in the future (27).

According to our model results, the cost of serplulimab significantly influenced the ICERs. Thus, these conditions could be ameliorated through charitable drug donations or price negotiations. Additionally, a donation policy for serplulimab currently exists. Patients who self-funded 600 mg of serplulimab were eligible to request an additional 600 mg of serplulimab as free assistance. Furthermore, if patients self-funded another 600 mg of serplulimab, they would continue to receive free medication until disease progression. The total duration of assistance would not exceed 2 years. According to the current drug donation policy, it was discovered that the ICER was 96,535.81$/QALY, surpassing the WTP threshold.

Our study, however, has some limitations. First, there is uncertainty in estimating the utility of PFS and PD. The utility of PFS and PD as a model parameter was determined from a recent first-line treatment study of advanced ESCC. Second, we made some assumptions when calculating the cost of second-line treatment. We assumed that patients received chemotherapy or immunotherapy alone. Radiation therapy, and other treatment strategies were not taken into consideration. This may not be consistent with actual clinical practice, which may underestimate the costs. Thirdly, any model has inherent biases, and the Markov model is no exception. We assumed that the ESCC patients were homogeneous in terms of their characteristics and preferences in the Markov model, which may deviate from the complex individual situations in reality. Finally, the costs of treatment for AEs in this study may have been underestimated, influencing the results. However, sensitivity analyses revealed that the costs of AEs had little effect on the ICERs.

## Conclusion

In conclusion, this is the first economic analysis of the addition of serplulimab to chemotherapy as a first-line treatment for PD-L1-positive patients with advanced ESCC using a Markov model, which presents evidence that can inform the development of relevant medical insurance policies and clinical decision-making.

## Data availability statement

The original contributions presented in the study are included in the article/supplementary material. Further inquiries can be directed to the corresponding author.

## Author contributions

Contributions: (I) Conception and design: All authors; (II) Administrative support: FW, NS; (III) Provision of study materials or patients: HZ, JL; (IV) Collection and assembly of data: HZ, JL; FW; (V) Data analysis and interpretation: HZ, JL; FW; (VI) Manuscript writing: All authors; (VII) Final approval of manuscript: All authors.

## References

[1] Sung H Ferlay J Siegel RL Laversanne M Soerjomataram I Jemal A. Global cancer statistics 2020: GLOBOCAN estimates of incidence and mortality worldwide for 36 cancers in 185 countries. CA: Cancer J Clin (2021) 71(3):209–49. doi: 10.3322/caac.21660 33538338

[2] Li J Xu J Zheng Y Gao Y He S Li H. Esophageal cancer: Epidemiology, risk factors and screening. Chin J Cancer Res (2021) 33(5):535–47. doi: 10.21147/j.issn.1000-9604.2021.05.01 PMC8580797 34815628

[3] Thrumurthy SG Chaudry MA Thrumurthy SSD Mughal M. Oesophageal cancer: risks, prevention, and diagnosis. BMJ (2019) 366:l4373. doi: 10.1136/bmj.l4373 31289038

[4] Wang J-N Che Y Yuan Z-Y Lu Z-L Li Y Zhang Z-R. Acetyl-macrocalin B suppresses tumor growth in esophageal squamous cell carcinoma and exhibits synergistic anti-cancer effects with the Chk1/2 inhibitor AZD7762. Toxicol Appl Pharmacol (2019) 365:71–83. doi: 10.1016/j.taap.2019.01.005 30633885

[5] Kitagawa Y Uno T Oyama T Kato K Kato H Kawakubo H. Esophageal cancer practice guidelines 2017 edited by the Japan Esophageal Society: part 1. Esophagus (2019) 16(1):1–24. doi: 10.1007/s10388-018-0641-9. 30171413 PMC6510883

[6] Mao C Zeng X Zhang C Yang Y Xiao X Luan S. Mechanisms of pharmaceutical therapy and drug resistance in esophageal cancer. Front Cell Dev Biol (2021) 9. doi: 10.3389/fcell.2021.612451 PMC7905099 33644048

[7] Sun JM Shen L Shah MA Enzinger P Adenis A Doi T. Pembrolizumab plus chemotherapy versus chemotherapy alone for first-line treatment of advanced oesophageal cancer (KEYNOTE-590): a randomised, placebo-controlled, phase 3 study. Lancet (2021) 398(10302):759–71. doi: 10.1016/S0140-6736(21)01234-4 34454674

[8] Doki Y Ajani JA Kato K Xu J Wyrwicz L Motoyama S. Nivolumab combination therapy in advanced esophageal squamous-cell carcinoma. N Engl J Med (2022) 386(5):449–62. doi: 10.1056/NEJMoa2111380 35108470

[9] Luo H Lu J Bai Y Mao T Wang J Fan Q. Effect of camrelizumab vs placebo added to chemotherapy on survival and progression-free survival in patients with advanced or metastatic esophageal squamous cell carcinoma: the ESCORT-1st randomized clinical trial. Jama (2021) 326(10):916–25. doi: 10.1001/jama.2021.12836 PMC8441593 34519801

[10] Wang ZX Cui C Yao J Zhang Y Li M Feng J. Toripalimab plus chemotherapy in treatment-naïve, advanced esophageal squamous cell carcinoma (JUPITER-06): A multi-center phase 3 trial. Cancer Cell (2022) 40(3):277–88.e3. doi: 10.1016/j.ccell.2022.02.007 35245446

[11] Lu Z Wang J Shu Y Liu L Kong L Yang L. Sintilimab versus placebo in combination with chemotherapy as first line treatment for locally advanced or metastatic oesophageal squamous cell carcinoma (ORIENT-15): multicentre, randomised, double blind, phase 3 trial. Bmj (2022) 377:e068714. doi: 10.1136/bmj-2021-068714 35440464 PMC9016493

[12] Shen L Kato K Kim SB Ajani JA Zhao K He Z. Tislelizumab versus chemotherapy as second-line treatment for advanced or metastatic esophageal squamous cell carcinoma (RATIONALE-302): A randomized phase III study. J Clin Oncol (2022) 40(26):3065–76. doi: 10.1200/JCO.21.01926 PMC9462531 35442766

[13] Song Y Zhang B Xin D Kou X Tan Z Zhang S. First-line serplulimab or placebo plus chemotherapy in PD-L1-positive esophageal squamous cell carcinoma: a randomized, double-blind phase 3 trial. Nat Med (2023) 29(2):473–82. doi: 10.1038/s41591-022-02179-2 PMC9941045 36732627

[14] Hoyle MW Henley W. Improved curve fits to summary survival data: application to economic evaluation of health technologies. BMC Med Res Method (2011) 11:1–14. doi: 10.1186/1471-2288-11-139 PMC3198983 21985358

[15] Liu Q Luo X Peng L Yi L Wan X Zeng X. Nivolumab versus docetaxel for previously treated advanced non-small cell lung cancer in China: A cost-effectiveness analysis. Clin Drug Investig (2020) 40(2):129–37. doi: 10.1007/s40261-019-00869-3 PMC6989620 31679121

[16] Eichler HG Kong SX Gerth WC Mavros P Jonsson B. Use of cost-effectiveness analysis in health-care resource allocation decision-making: how are cost-effectiveness thresholds expected to emerge? Value Health (2004) 7(5):518–28. doi: 10.1111/j.1524-4733.2004.75003.x 15367247

[17] Lu S Ye M Ding L Tan F Fu J Wu B. Cost-effectiveness of gefitinib, icotinib, and pemetrexed-based chemotherapy as first-line treatments for advanced non-small cell lung cancer in China. Oncotarget (2017) 8(6):9996. doi: 10.18632/oncotarget.14310 28036283 PMC5354787

[18] Zhang Q Wu P He X Ding Y Shu Y. Cost-effectiveness analysis of camrelizumab vs. placebo added to chemotherapy as first-line therapy for advanced or metastatic esophageal squamous cell carcinoma in China. Front Oncol (2021), 11:790373. doi: 10.3389/fonc.2021.790373 34926306 PMC8671697

[19] Global burden of 369 diseases and injuries in 204 countries and territories, 1990-2019: a systematic analysis for the Global Burden of Disease Study 2019. Lancet (2020) 396(10258):1204–22. doi: 10.1016/S0140-6736(20)30925-9 PMC7567026 33069326

[20] Qiu H Cao S Xu R. Cancer incidence, mortality, and burden in China: a time-trend analysis and comparison with the United States and United Kingdom based on the global epidemiological data released in 2020. Cancer Commun (Lond) (2021) 41(10):1037–48. doi: 10.1002/cac2.12197 PMC8504144 34288593

[21] Li Y Xu J Gu Y Sun X Dong H Chen C. The disease and economic burdens of esophageal cancer in China from 2013 to 2030: dynamic cohort modeling study. JMIR Public Health Surveill (2022) 8(3):e33191. doi: 10.2196/33191 34963658 PMC8928052

[22] Cao X Cai H Li N Zheng B Zheng Z Liu M. First-line nivolumab plus ipilimumab or chemotherapy versus chemotherapy alone for advanced esophageal cancer: a cost-effectiveness analysis. Ther Adv Med Oncol (2022) 14:17588359221122733. doi: 10.1177/17588359221122733 36147862 PMC9486256

[23] Zhu Y Liu K Ding D Zhou Y Peng L. Pembrolizumab plus chemotherapy as first-line treatment for advanced esophageal cancer: A cost-effectiveness analysis. Adv Ther (2022) 39(6):2614–29. doi: 10.1007/s12325-022-02101-9 35394255

[24] Shen J Du Y Shao R Jiang R. First-line sintilimab plus chemotherapy in locally advanced or metastatic esophageal squamous cell carcinoma: A cost-effectiveness analysis from China. Front Pharmacol (2022) 13:967182. doi: 10.3389/fphar.2022.967182 36569294 PMC9767976

[25] Liu L Wang L Chen L Ding Y Zhang Q Shu Y. Cost-effectiveness of sintilimab plus chemotherapy versus chemotherapy alone as first-line treatment of locally advanced or metastatic oesophageal squamous cell carcinoma. Front Immunol (2023) 14:1092385. doi: 10.3389/fimmu.2023.1092385 36756110 PMC9899904

[26] The L. Cancer drugs in China: affordability and creativity. Lancet (2018) 391(10133):1866. doi: 10.1016/S0140-6736(18)31034-1 29781431

[27] Liu GG Wu J He X Jiang Y. Policy updates on access to and affordability of innovative medicines in China. Value Health Reg Issues (2022) 30:59–66. doi: 10.1016/j.vhri.2021.12.003 35235902

